# Pimpinellin Inhibits Collagen-induced Platelet Aggregation and Activation Through Inhibiting Granule Secretion and PI3K/Akt Pathway

**DOI:** 10.3389/fphar.2021.706363

**Published:** 2021-07-22

**Authors:** Gang Liu, Zhaowei Yuan, Xiaoyun Tian, Xiuqin Xiong, Fang Guo, Zihan Lin, Zhen Qin

**Affiliations:** ^1^ School of Basic Medical Sciences, Guizhou Medical University, Guiyang, China; ^2^ Guizhou Provincial Engineering Technology Research Center for Chemical Drug R&D, Guizhou Medical University, Guiyang, China; ^3^ Guizhou Provincial Key Laboratory of Pathogenesis and Drug Research on Common Chronic Diseases, Guizhou Medical University, Guiyang, China

**Keywords:** pimpinellin, platelet, granule secretion, hemostasis, PI3K/AKT signalling

## Abstract

Pimpinellin is a coumarin-like compound extracted from the root of *Toddalia asiatica*. Its effects on platelet function has not been investigated. This study found that pimpinellin pretreatment effectively inhibited collagen-induced platelet aggregation, but did not alter ADP- and thrombin-induced aggregation. Platelets pretreated with pimpinellin showed reduced α granule (CD62) level and secretion of dense granule (ATP release). Pimpinellin-treated platelets also exhibited decreased clot reaction and TxB2 production. Pimpinellin pretreatment suppressed adhesion and spreading of human platelets on the fibrinogen coated surface. Analysis of tail bleeding time of mice administered with pimpinellin (40 mg/kg) revealed that pimpinellin did not change tail bleeding time significantly, number of blood cells, and APTT and PT levels. Pimpinellin inhibited collagen-induced *ex vivo* aggregation of mice platelets. Immunoblotting results showed that pimpinellin suppressed collagen-induced phosphorylation of PI3K-Akt-Gsk3β and PKC/MAPK in platelets.

## Introduction

Platelets play a central role in thrombosis and hemostasis. In addition, they are the smallest biologically active blood cells and are the core regulators of the important balance between thrombosis and bleeding in the blood circulation (Koupenova et al., 2017a). Platelets also play an important role in many important pathophysiological processes, such as atherosclerosis (Davi and Patrono, 2007), immune response (Rondina et al., 2013), infection, tumor progression, and metastasis (Jorgensen, 2006; Lam et al., 2017). Thrombus formation is caused by several dysregulated factors such as endothelial cells (ECs) activation, lipoprotein oxidation, and immune response (Faber et al., 2009). Platelet activation is initiated by the binding of various ligands to their surface receptors, which triggers an intracellular signaling cascade. This leads to platelets’ change in shape, the release of particle content, synthesis and release of thromboxane A2, and thrombus formation (Gibbins, 2004). An important pathological basis of thromboembolic diseases is the abnormal activation of platelets (Koupenova et al., 2017b), and antiplatelet therapy is an important way to prevent and treat thrombotic diseases.

There are three classes of antithrombotic drugs: anticoagulant drugs, thrombolytic drugs, and antiplatelet drugs (Gallus and Hirsh, 1976). Despite significant advances in understanding the nature of the thrombotic process and the use of drugs such as urokinase, aspirin, or clopidogrel, the therapeutic efficacy and prognosis remain limited (Michelson, 2008). For example, aspirin or clopidogrel may cause several serious side effects, including liver damage, renal insufficiency, and gastric bleeding. Most of the currently known coumarins are reported to have strong antiplatelet activity and anti-fungal efficacy (Zaragoza et al., 2016; Hu et al., 2017). Warfarin inhibits platelet aggregation and 5-hydroxytryptamine (5-HT) secretion in a concentration-dependent manner (Nilsson et al., 2019); Isofraxidin is a natural coumarin that significantly inhibits ADP and AA-induced platelet aggregation (Jin et al., 2020); Columbianadin is the main coumarin isolated from *Angelica pubescens* Maxim. *f. biserrata* Shan *et* Yuan, and it effectively inhibits collagen-induced platelet aggregation (Hou et al., 2020). *Toddalia asiatica Lam* roots functions in dispelling wind and pain, dispersing blood stasis, stopping bleeding, hemostasis and anti-inflammatory activities (Kariuki et al., 2013). Besides, it is also used anti-cancer, antimicrobial, and antidiabetic (Stephen et al., 2012; Li et al., 2018).

Pimpinellin is a major coumarin-like compound obtained from *Toddalia asiatica Lam* (Liu et al., 2014). However, the antiplatelet and antithrombotic effects of pimpinellin have not been reported. Therefore, in this study, we investigated the antiplatelet and antithrombotic effects of pimpinellin. We investigated the effect of pimpinellin on platelet function in response to various agonists, as well as its influence on intracellular signaling pathways. In the present study, we demonstrated that pimpinellin is a potent inhibitor of platelet function with potential as an antiplatelet agent for the prevention of thrombotic disease.

## Materials and Methods

### Materials

Pimpinellin (HPLC≥98%) was purchased from Shanghai Macklin Technology Corp (Macklin, Shanghai, China). Luciferin and collagen were purchased from the Chrono-Log Corp (Havertown, PA, United States). Thrombin, ADP, and FITC-Phalloidin were purchased from Sigma (St. Louis, MO, United States). PAC-1 and CD62P (P-selectin) antibody were purchased from BD Biosciences (San Jose, CA, United States). Antibody against total-p85, total-Akt, total-GSK3β, total-p38, total-ERK, total-JNK, total-Syk, total-SLP76, total-PLCγ2, and phospho-Akt (Ser473) were from Santa Cruz Biotechnology (Santa Cruz, CA, United States). Antibody for phospho-PI3K (p85/p55), phospho-GSK3β, phospho-p38, phospho-ERK, phospho-JNK, phosphor-SLP76, phosphor-PLCγ2, and phospho-PKC substrates was from Cell Signaling Technology (Beverly, MA, United States). The phospho-Syk antibody was obtained from GeneTex International Corp (GeneTex, Taiwan, China). GAPDH was purchased from Proteintech Group, Inc (Proteintech, IL, United States). The thromboxane B2 and cyclic adenosine monophosphate (AMP) enzyme immunoassay (EIA) kits were from Cayman (Ann Arbor, MI, United States); ECL Western blotting detection reagent was obtained from Millipore Corp (Millipore, MA, United States). Pimpinellin was dissolved in DMSO and stored at −20°C.

### Animal

C57BL/6 mice were maintained at the Animal Experiment Center of Guizhou Medical University. All mice were handled, monitored, and executed in accordance with the approved guidelines. Besides, they were housed in a controlled environment (humidity 40%–60%, 24 ± 2°C) with free access to water and food, and domesticated for 1 wk before experimental manipulation. All animal experiments were approved by the Animal Care Welfare Committee of Guizhou Medical University.

Mice were randomly divided into four groups, blank group, aspirin group (100 mg/kg), and pimpinellin group (40 and 100 mg/kg). For mice, the dose was administered by gavage for 1 wk, followed by tail bleeding time and *ex vivo* platelet aggregation tests.

### Platelet Preparation

Human blood was obtained from healthy volunteers in accordance with the Declaration of Helsinki guidelines and the Ethics Committee of Guizhou Medical University. Human platelet-rich plasma (PRP) and washed human platelets were prepared as previously described (Vaiyapuri et al., 2015). Blood was collected from the elbow vein of a healthy volunteer, then the whole blood was centrifuged at 200 × g for 10 min, the supernatant was aspirated and diluted with 1×Tyrodes Buffer; PGE1 (50 ng/ml) and Apyrase (0.1 U/ml) were added and centrifuged at 700 × g for 10 min to obtain platelet clumps, which were diluted with 1×Tyrodes Buffer and repeated 2 times. The platelets were resuspended in 1×Tyrodes buffer to a final concentration of 3.0 × 10^8^/ml.

### Platelet Aggregation and ATP Release

An aggregometer was used to measure platelet aggregation and secretion. The washed human platelets were adjusted to 3.0 × 10^8^/ml with modified Tyrodes buffer and stimulated with ADP, collagen, and thrombin. Luciferase reagent was added to the platelet suspension to monitor platelet aggregation and adenosine triphosphate (ATP) secretion. Before stimulation, platelets were incubated with pimpinellin (10 and 20 μM) at 37°C for 10 min.

### Determination of Lactate Dehydrogenase (LDH)

Cytotoxic effects were examined by measuring the level of lactate dehydrogenase (LDH). Washed platelets were pre-incubated with pimpinellin (10 and 20 μM) at 37°C for 10 min. Finally, an aliquot (20 μl) of the supernatant was assessed using the LDH assay kit (Nanjing Jiancheng Bioengineering Institute, Jiangsu, China). The maximal value of LDH was observed in Triton-treated platelets.

### Flow Cytometry

CD62P expression in platelets and PAC-1 binding to activated αIIbβ3 was determined using the FACScan flow cytometer. Unstirred washed platelets (5.0 × 10^7^/ml) were incubated with 20 μM pimpinellin or vehicle at 37°C for 10 min. Then, platelets were activated with agonist CRP (1 μg/ml) for 5 min. The washed platelets were pre-incubated with fluorescein isothiocyanate (FITC)-conjugated PAC-1 antibody, FITC-conjugated CD62P antibody, or isotype control antibody. Flow cytometry was performed after 15 min of treatment in a dark room at room temperature.

### Platelet Spreading on Fibrinogen

The spreading of platelets on the fibrinogen-coated surface was carried out as described previously (Yue et al., 2016). The adhered platelets were observed with a fluorescence microscope. The image was acquired with a Nikon camera. ImageJ software was used to analyze the number of adherent platelets and the average spreading area.

### Clot Retraction

Washed platelets were incubated with vehicle or pimpinellin (10, 20 and 40 μM) at 37°C for 10 min and the reaction mixture was transferred to a glass tube for observation. Trigger clot retraction by adding thrombin (0.4 U/ml) and proceed at 37°C. Take pictures after 30 min to observe the phenomenon.

### Thromboxane B_2_ (TxB_2_) Assay and Measurement of Cyclic AMP (cAMP) Levels

Pre-incubate platelets (3 × 10^8^/ml) with vehicle or pimpinellin (10 and 20 μM) for 10 min, and then treated with collagen. Ethylenediaminetetraacetic acid (EDTA, 2 mM) was added to the platelet suspension. The TxB_2_ and circulating AMP levels were measured for each specimen using a Cayman ELISA kit, centrifugation at 12,000 rpm for 10 min.

### Immunoblotting

Washed platelets were incubated with pimpinellin for 10 min, and stimulated with or without an agonist for 3 min. A similar volume of 2 × Lysis Buffer was added to the reaction to lyse the aggregated platelets. The lysate was prepared, analyzed by SDS-PAGE, electro-transferred to PVDF membrane. Then it was blocked with 5% (w/v) BSA and detected with a primary antibody. The membrane was rinsed with TBST and incubated with appropriate secondary antibodies. Western blotting was carried out as described previously (Liu et al., 2016).

### Tail Bleeding Assay

The mice were randomly divided into four groups; aspirin group (100 mg/kg), pimpinellin group (40 and 100 mg/kg), and control group (0.5% CMC-Na). To calculate the amount of gavage, mice were weighed and dose administration was performed by gavage daily for 1 wk. Mice were anesthetized before the tail was transected 5 mm from the tip using a sterile scalpel. A bleeding assay was performed by excision of a tail from the tip, and the time taken for cessation of bleeding was recorded.

### Hematology Assessment

Two hours after administration of the last gavage, mice blood was taken after anesthesia, and centrifuged at 1200 rpm for 10 min, and the supernatant was used to detect activated partial thromboplastin time (APTT) and prothrombin time (PT). Using standard mice parameters, complete hematology analysis was performed using an automatic cell counter (Mindray; BC-5130).

### *Ex vivo* Platelet Aggregation

One week after the last gavage, washed platelets (3.0 × 10^8^/ml) were prepared and stimulated with collagen. The platelet aggregation rate is measured with an aggregator, and the result is recorded by the aggregolink software.

### Statistical Analysis

All the data were generated from at least three independent experiments, with data presented as mean ± standard error of the mean (SEM). All data were tested for significance using one-way ANOVA with Bonferroni’s *post hoc* test, *p* < 0.05 was considered statistically significant.

## Results

### Pimpinellin Inhibits Platelet Aggregation and ATP Secretion Induced by Collagen

**FIGURE 1:**
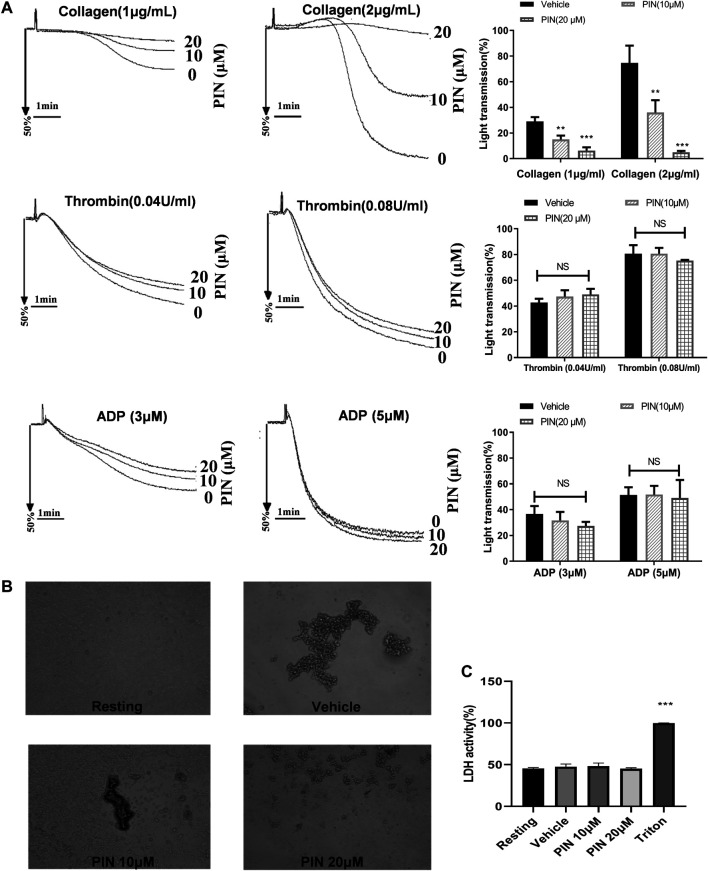
Effects of pimpinellin on platelet aggregation and LDH release. **(A)** The washed human platelets (3 × 10^8^/ml) were incubated with different concentrations of pimpinellin (10 and 20 μM) or the vehicle group for 5 min. Stimulated platelet aggregation with collagen (1 and 2 μg/ml), Thrombin (0.04 and 0.08 U/ml), ADP (3 and 5 μM). **(B)** Fixed and observed untreated platelets or platelets treated with vehicle or pimpinellin (10 and 20 μM) and collagen 2μg/mL. Values are the average of three independent experiments ± S.E.M. **(C)** To assess the cytotoxicity, platelets were pre-incubated with 0.1% DMSO (vehicle), or pimpinellin (10 and 20 μM) for 10 min, and 20 μl of the supernatant from the aliquots was precipitated on an LDH-assay kit. Data are presented as the mean ± SEM (*n* = 9). One-way ANOVA followed by Bonferroni *post-hoc* analysis of the data. (**p < 0.01, ***p < 0.001 compared with the vehicle group, #*p* < 0.05 compared with the PIN 10 μM group, *NS* means no significance.).

We examined the effect of pimpinellin on platelet aggregation induced by different agonists. Pimpinellin was found to inhibit platelet aggregation induced by collagen (Figure 1A). Collagen (2 μg/ml) in stimulated platelet aggregation rate was reduced by pimpinellin (10 and 20 μM) from 74 ± 8% to 36 ± 5% and 5 ± 1% (*n* = 3, *p* < 0.001). The 50% inhibitory concentration (IC50) of pimpinellin on collagen (2 μg/ml)-induced platelet aggregation was 13.6 μM. Pimpinellin exhibited a inhibition of collagen-stimulated platelet aggregation but it had no effect on thrombin (0.04 and 0.08 U/ml), ADP (3 and 5 μM)-induced platelet aggregation. Meanwhile, pimpinellin did not affect ADP-induced platelet aggregation in PRP (Supplementary Figure S1). The anti-platelet effect of pimpinellin can also be confirmed by microscopic observation that pimpinellin reduced the formation of platelet aggregates (Figure 1B).

**FIGURE 2:**
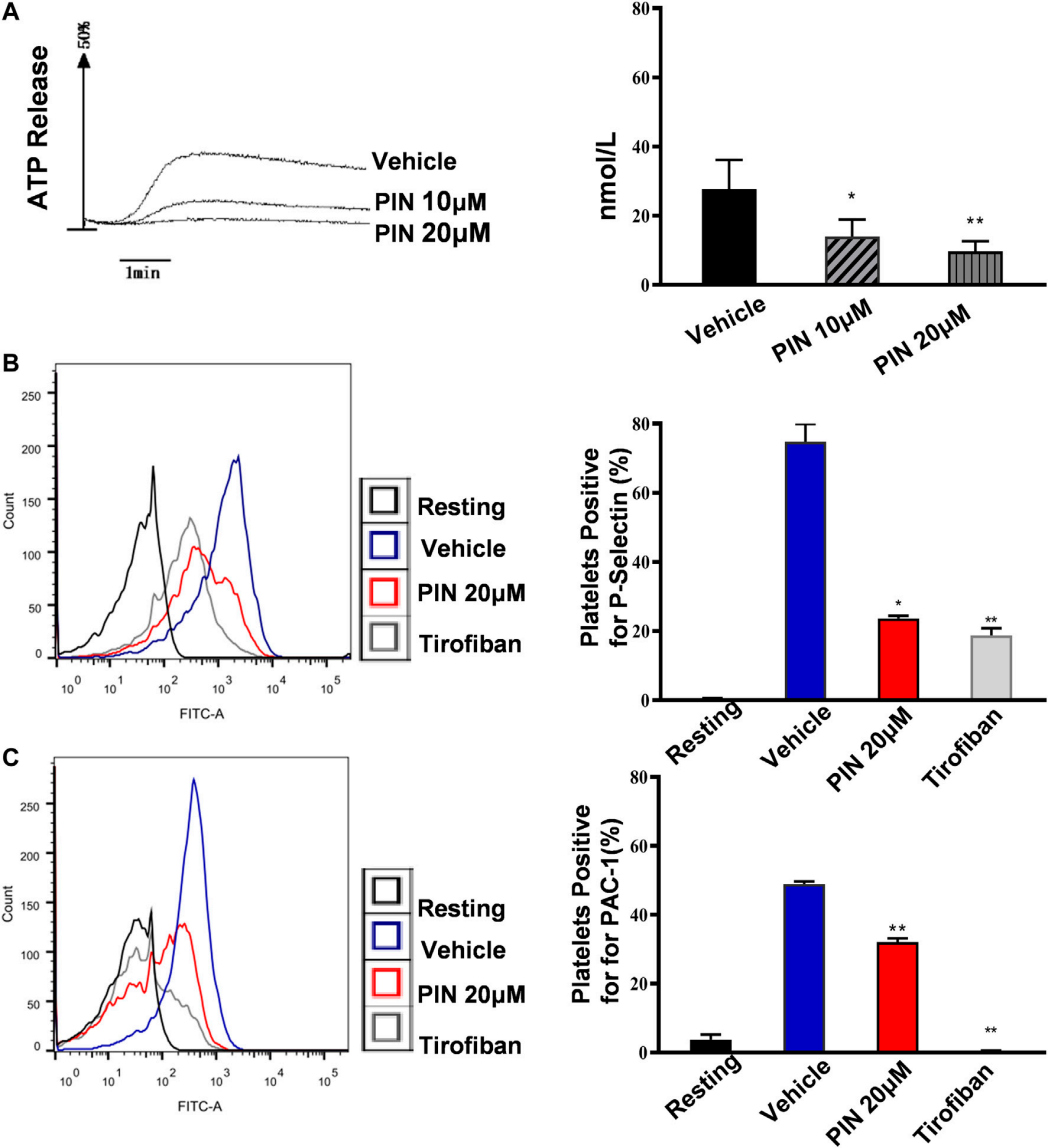
Pimpinellin inhibited platelet ATP release, P-selectin expression and PAC-1. **(A)** Washed platelets were treated with pimpinellin (10 and 20 μM) at 37°C for 5 min and ATP release was measured after stimulation with collagen (1 μg/ml) using a Lumi-Aggregometer. One-way ANOVA followed by Bonferroni *post-hoc* analysis of the data. (*p < 0.05, **p < 0.01; compared to the vehicle group). **(B**,**C)** Washed platelets (3.0  ×  10^8^/ml) were incubated with vehicle or pimpinellin (20 μM), Then CRP (1 μg/ml) was added to stimulate the expression of P-selection **(B)** and PAC-1 **(C)**. (resting control, black line; CRP-activated, blue line; pimpinellin 20 μM, red line; Tirofiban, brown line). Detection of P-selection, PAC-1 expression by flow cytometry (*n* = 3). Bar graphs show mean ± SEM (*n* = 3). *p < 0.05 and **p < 0.01 compared to the vehicle group.

A similar inhibitory effect on collagen-induced ATP release was also observed (Figure 2A). The data indicate that 20 μM pimpinellin can effectively inhibit the release of ATP. These results strongly suggested that pimpinellin suppressed platelet aggregation and ATP release.

### Effects of Pimpinellin on LDH Release

Pimpinellin (10 and 20 μM) did not significantly (*p* > 0.05) increase LDH activity (Figure 1C). This indicated that pimpinellin did not affect platelets permeability or induce platelet cytolysis.

### Pimpinellin Inhibits Platelet Activation

To further demonstrate that pimpinellin inhibits the release of platelet granules content, we examined the expression of CRP (1 μg/ml)-induced platelet surface P-selectin expression. Compared with the control group without pimpinellin, pimpinellin evidently reduced CRP-stimulated P-selectin expression (Figure 2B).

PAC-1 is used to detect the activation level of human platelet integrin aIIbβ3. To determine whether pimpinellin could interfere with integrin αIIbβ3 activation, flow cytometry was used to assess PAC-1 binding. In the presence of pimpinellin (20 μM), PAC-1 binding was significantly reduced (*p* < 0.01) (Figure 2C).

### Pimpinellin Negatively Regulates Integrin αIIbβ3-Mediated Outside-in Signaling

**FIGURE 3:**
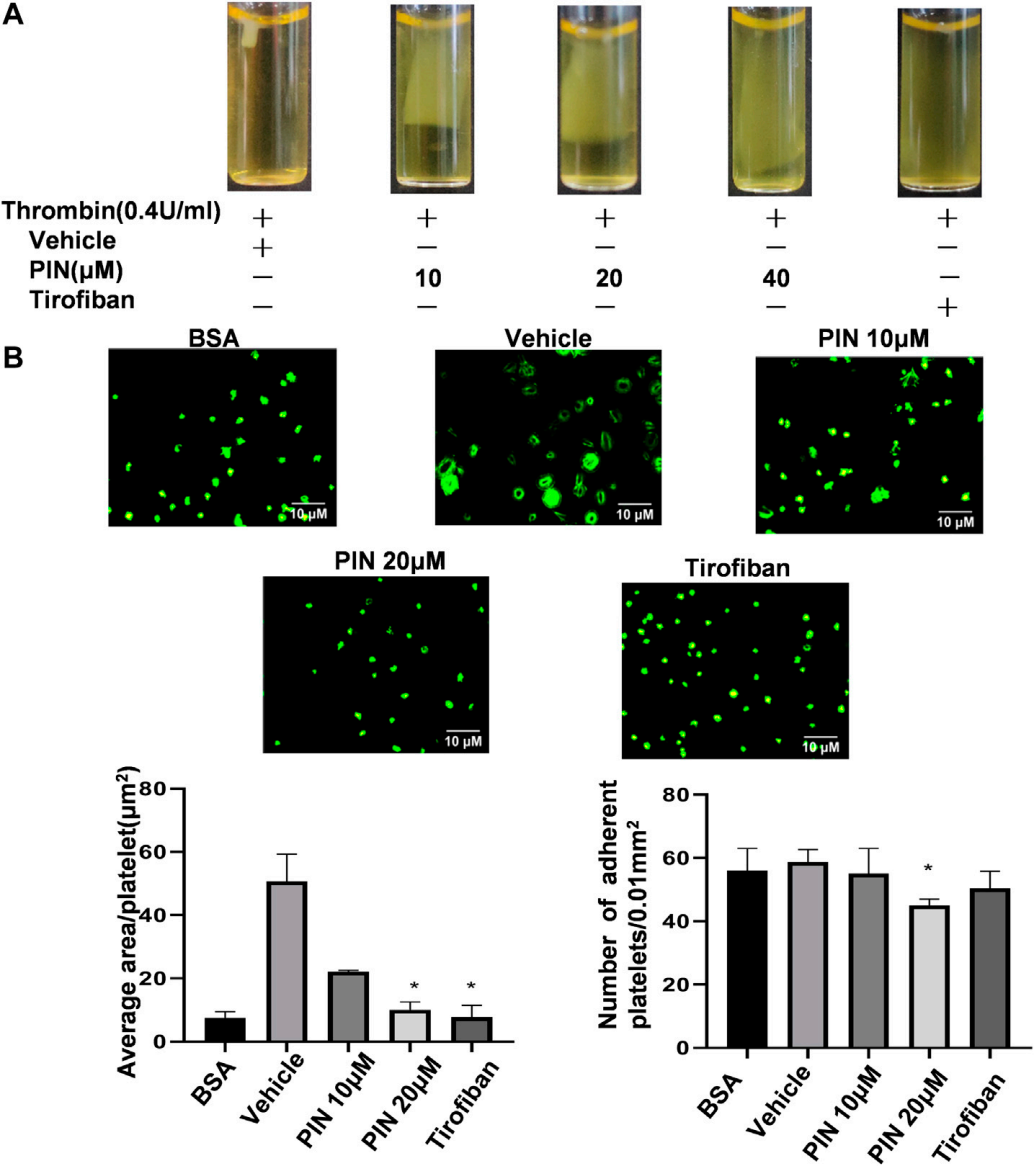
The effect of pimpinellin on clot retraction and platelet spreading on fibrinogen surfaces. **(A)** Human platelet-rich plasma were treated with pimpinellin (10, 20 and 40 μM) at 37°C for 5 min. The clot retraction was triggered by adding thrombin (0.4 U/ml) and proceeding at 37°C. The clot retraction was photographed after 30 min. **(B)** Human washed platelets (1.0 × 10^7^/ml) were pretreated with pimpinellin (10 and 20 μM) or vehicle at 37°C for 5 min, and then allowed to adhere to the fibrinogen-coated glass slide. Platelets were spread on a slide coated with fibrinogen at 37°C for 60 min, after which spreading was stopped by fixing in 4% paraformaldehyde. The platelet images were obtained by fluorescent microscopy. Representative images were obtained from three similar experiments. Data are presented as the mean ± SEM (*n* = 3). *p < 0.05 and **p < 0.01 compared to the vehicle group.

As shown in Figure 3A, clot retraction was reduced significantly in the presence of pimpinellin (10, 20, and 40 μM) at 30 min compared with the vehicle group. We selected the integrin αIIbβ3 antagonist tirofiban as a positive control (Huang et al., 2019) and found that tirofiban could significantly inhibited clot retraction.

Next, we examined the spreading of platelets on the surface of fibrinogen in the presence of pimpinellin at different concentrations (10 and 20 μM). As shown in Figure 3B, pimpinellin (20 μM) significantly inhibited platelet spreading and reduced singlel platelet surface coverage area from 50.0 ± 8.6 μm^2^ to 9.89 ± 0.64 μm^2^, respectively. Compared to the vehicle group, pimpinellin (20 μM) inhibited platelet adhesion from 55 ± 8 platelets/0.01 mm^2^ to 38 ± 1 platelets/0.01 mm^2^.

These data suggested that the outside-in signaling pathway that regulates the coordinated process of clot retraction and platelet spreading on fibrinogen *via* integrin αIIbβ3 was influenced by pimpinellin.

### Effects of Pimpinellin on Collagen-induced Platelet TxB_2_ Production and cAMP Levels

**FIGURE 4:**
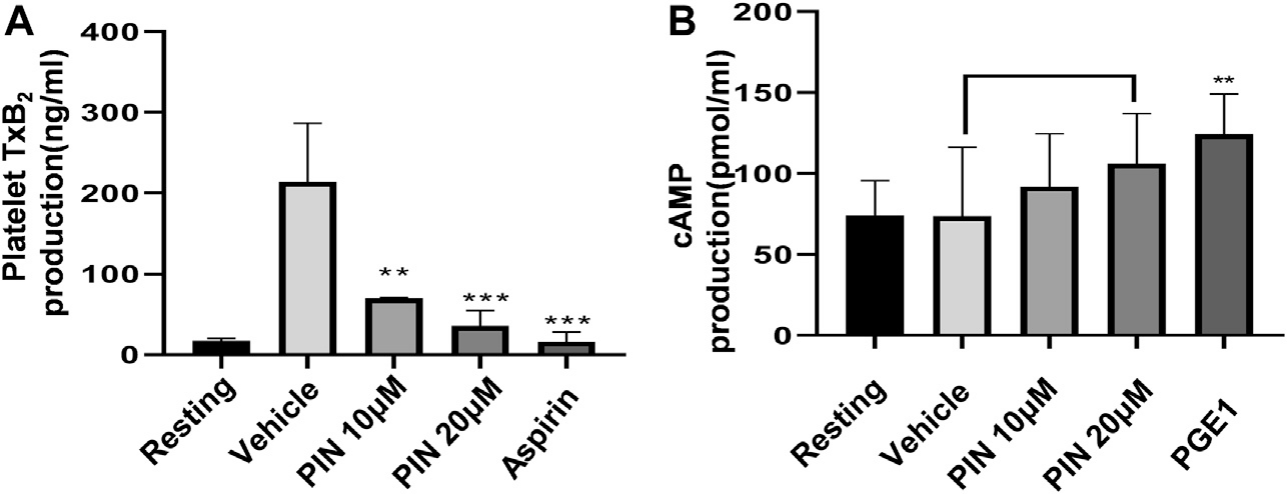
Effects of pimpinellin on cAMP levels, and TxB_2_ production. **(A)** We pre-incubated washed human platelets (3 × 10^8^/ml) with vehicle, different concentrations of pimpinellin, or aspirin (100 μM) for 10 min, after which the platelets were stimulated with collagen (1 μg/ml). TxB_2_ production levels were evaluated by measuring the OD values. Results were expressed as TxB_2_ production (ng/ml). **(B)** Washed human platelets (3 × 10^8^/ml) were pre-incubated with the vehicle, different concentrations of pimpinellin or PGE1 (50 ng/ml) for 10 min, after which they were stimulated with collagen. After 5 min, the supernatant was obtained by centrifugation. The effect of pimpinellin on cAMP levels was determined by detecting OD value. Data are mean ± SEM values (*n* = 4). *p < 0.05, **p < 0.01, ***p < 0.001 compared to the vehicle group.

To investigate the possible mechanism of the anti-platelet effect of pimpinellin, the effects on TxB_2_ formation were evaluated. Compared with collagen-activated platelets, resting platelets produce less TxB_2._ Pimpinellin (10 and 20 μM) significantly (*p* < 0.001) inhibited the formation of platelet TxB_2_ stimulated by collagen (1 μg/ml) (Figure 4A).

We determined the cAMP levels in pimpinellin-pretreated platelets interacting with different reagents. Our findings showed that pimpinellin does not affect cAMP production compared with resting washed platelets (Figure 4B).

### Pimpinellin Inhibits Signaling Pathways of Platelet Activation Induced by Collagen

**FIGURE 5:**
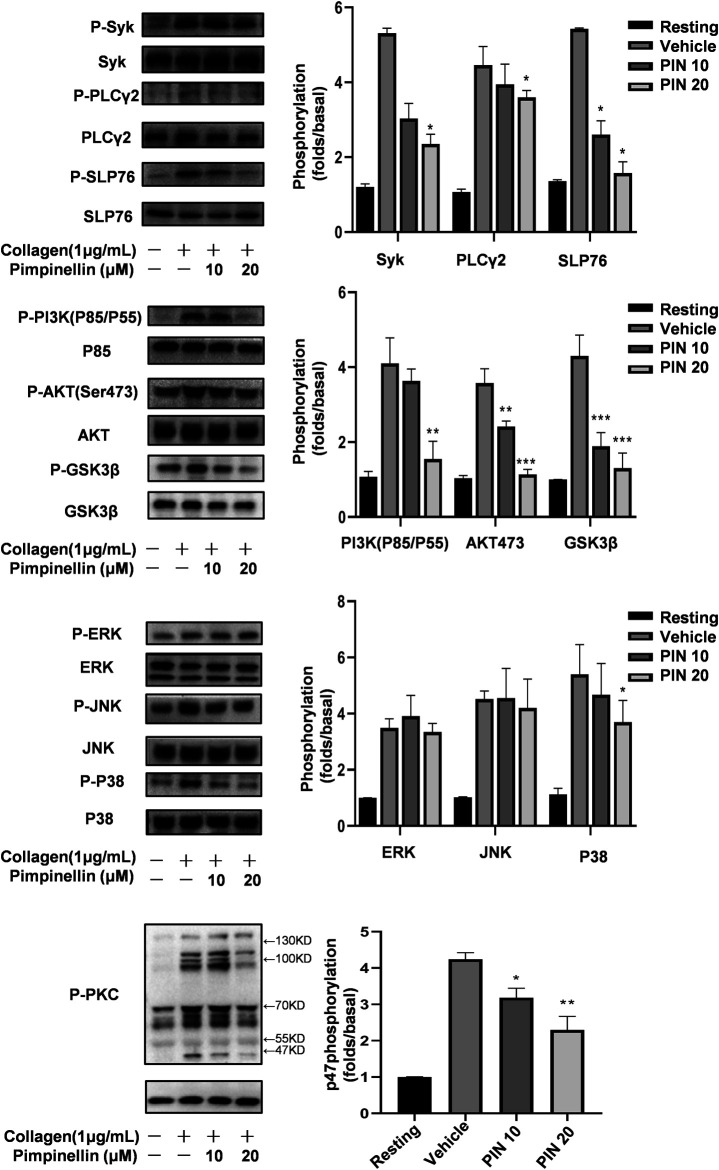
Effect of pimpinellin on collagen-induced signal transduction. Washed platelets were stimulated with 2 μg/ml collagen (3.0 × 10^8^/ml) and then lysed with cell lysis buffer. Syk, PLCγ2, SLP76, JNK, ERK, P-38, p85, Akt, GSK3β, and PKC phosphorylation levels were detected with relevant antibodies. Band density was analyzed using the ImageJ software. Data are presented as the mean ± SEM (*n* = 3). *p < 0.05 and **p < 0.01 compared to the vehicle group; *NS* means no significance.

To explore the molecular mechanism of pimpinellin inhibition of platelet aggregation, platelet lysates were obtained from platelets pre-incubated with pimpinellin (10 and 20 μM) for 5 min, they were then lysed for 5 min after collagen stimulated. Western blot analysis showed that Syk, SLP76, PLCγ2, PI3K (p85/p55), Akt and GSK3β phosphorylation were inhibited by pimpinellin. We concluded that pimpinellin may negatively regulated of Syk-SLP76-PLCγ2-PI3K-Akt-GSK3β signaling (Figure 5).

We evaluated the MAPK signaling molecules including p38 MAPK, JNKs, and ERKs. Our findings showed that pimpinellin inhibited the phosphorylation of p38, but not ERK or JNK. Therefore, the inhibition of p38 signaling may be involved in the mechanism of pimpinellin-mediated inhibition (Figure 5).

Stimulation of platelets with collagen induces the activation of protein kinase C (PKC) and subsequent phosphorylation of p47 proteins. When collagen was added to human platelets, 47 kDa (p47) was predominately phosphorylated compared to resting platelets. Data shows the inhibition of PKC activation in platelets following incubation with different concentrations of pimpinellin (Figure 5).

### Exogenous ADP Normalizes Pimpinellin Inhibition of Platelet Aggregation

**FIGURE 6:**
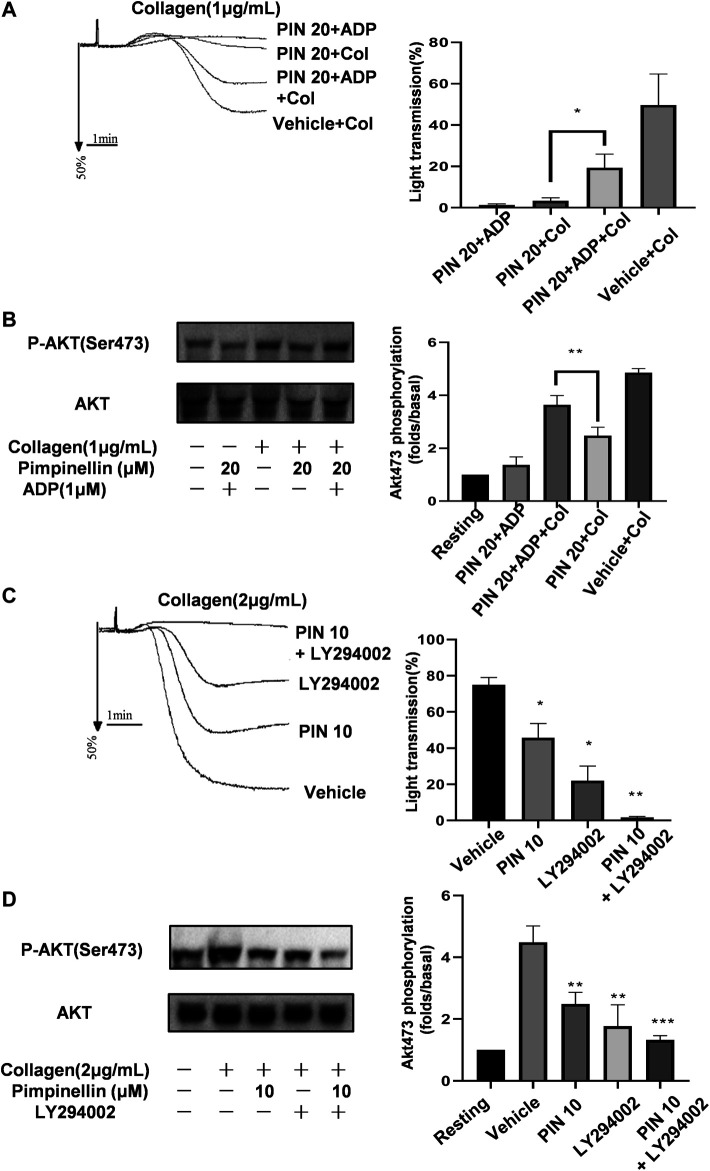
Effects of pimpinellin on platelet aggregation in the presence of ADP or LY294002. **(A)** Washed platelets (3 × 10^8^/ml) were pretreated with 20 μM pimpinellin, 1 μM ADP or both for 10 min after which they were stimulated with collagen (1 μg/ml). Platelet aggregation rates were calculated in at least three independent experiments and the results are shown as mean ± SEM (*p < 0.05 compared with the PIN 20+Col group, PIN 20 means pimpinellin 20 μM, COL means collagen). **(B)** Lysates from each group were analyzed by immunoblotting to determine the Akt phosphorylation levels. **(C)** Washed platelets (3 × 10^8^/ml) were pre-incubated with 10 μM pimpinellin, 2 μM LY294002 or both for 10 min, after which they were stimulated with 2 μg/ml collagen. (*p < 0.05 and **p < 0.01 compared to the vehicle group, PIN 10 means pimpinellin 10 μM). **(D)** Lysates in each group were analyzed by immunoblotting to determine the Akt phosphorylation levels. Band density was calculated by the ImageJ software. Bar graphs represent the mean ± SEM (*n* = 3).

We supplemented pimpinellin-incubated platelets with exogenous ADP to verify whether it could restore the aggregation rate of pimpinellin-inhibited platelets to normal levels (Figure 6A). ADP (1 μM) is not enough to induce platelet aggregation, but reversed the inhibitory effect of pimpinellin-incubated platelets aggregation stimulated by collagen (1 μg/ml). And ADP restored the decreased phosphorylation of Akt (Figure 6B).

### Effect of LY294002 on Pinpinellin-treated Platelet Aggregation

PI3K/Akt signaling plays a central role in platelet activation and granule secretion. We previously found that pimpinellin significantly inhibited the phosphorylation of PI3K (p85/p55) and Akt. Besides, pimpinellin combined with the PI3K-specific inhibitor LY294002 significantly inhibited collagen-activated platelet aggregation and Akt phosphorylation (Figures 6C,D). These findings confirmed that the mechanism of pimpinellin inhibition of platelet activation involved the PI3K/Akt signaling pathway.

### Effect of Pimpinellin on Coagulation Function and Platelet Aggregation *ex vivo*

**FIGURE 7:**
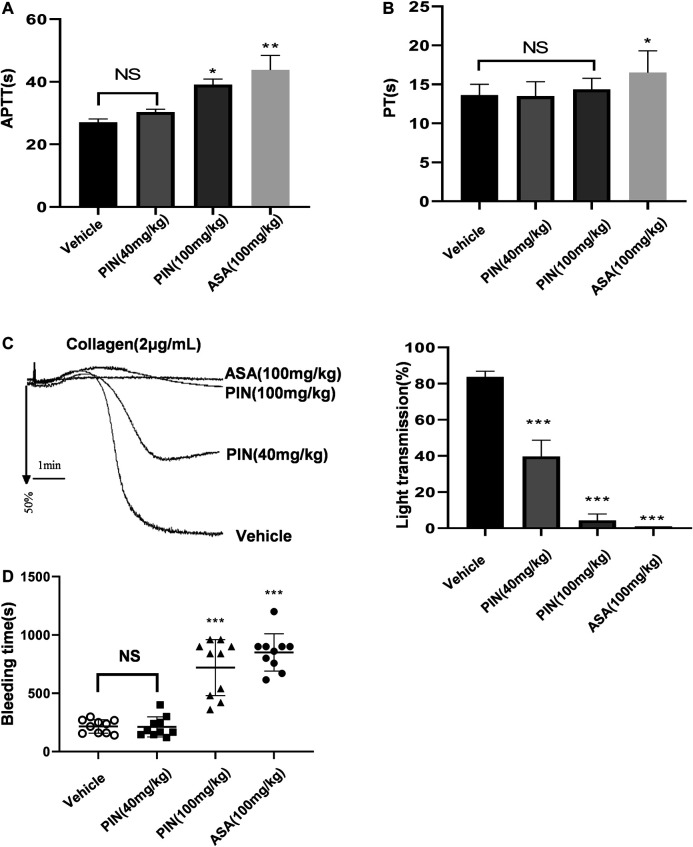
The effects of pimpinellin on coagulation in mice. **(A**,**B)** The effect of pimpinellin on the coagulation system was assessed by the APTT and PT. (*p < 0.05, **p < 0.01, *NS* means *p* > 0.05). **(C)** Representative experiments of platelet aggregation induced by collagen (2 μg/ml) in control mice and mice treated with pimpinellin or aspirin. Pimpinellin produced a inhibition of collagen-induced *ex vivo* aggregation in platelets. Statistics of aggregation rates (*n* = 3). Data are presented as mean ± SEM. *p < 0.05 compared to the vehicle group. **(D)** The tail tip was excised and soaked at 37°C for the bleeding test. Symbols represent the time to stop bleeding in individual animals and error bars represent the mean ± SEM (*n* = 10).

Compared with the control group, pimpinellin (40 mg/kg) did not affect the APTT levels, an indicator of endogenous coagulation pathway. However, pimpinellin (100 mg/kg) significantly prolonged the APTT in mice (*p* < 0.001) (Figure 7A), PT is mainly used to evaluate the exogenous coagulation pathway, pimpinellin did not affect the PT levels (Figure 7B). In this study, pimpinellin did not affect exogenous coagulation pathway, suggesting that the anticoagulation mechanism of high dose pimpinellin was mainly mediated by the endogenous coagulation system. In addition, pimpinellin (40 mg/kg) did not influence platelet count (PLT), mean platelet volume (MPV), white blood cell count (WBC) and red blood cell count (RBC) (Table 1).

**TABLE 1:** Effect of pimpinellin on coagulation index in mice.

Group	PLT (10^6^ ml^−1^)	MPV (μm^3^)	WBC (10^6^ ml)	RBC(10^9^ ml^−1^)
CMC-Na (0.5%)	587.800 ± 22.522	5.630 ± 0.096	1.815 ± 0.236	5.556 ± 0.206
Aspirin (100 mg/kg)	562.100 ± 35.652	5.440 ± 0.048	1.536 ± 0.178	5.699 ± 0.202
PIN(40 mg/kg)	570.100 ± 33.581	5.460 ± 0.06	1.985 ± 0.239	5.498 ± 0.418
PIN(100 mg/kg)	721.400 ± 61.666^a^	5.670 ± 0.073	1.279 ± 0.135	5.492 ± 0.134

Data are presented as the mean ± SEM (*n* = 10). Mice were orally administered with CMC-Na, pimpinellin (40 and 100 mg/kg), and ASA for a week. Effect of pimpinellin on mouse platelet count (PLT)、mean platelet volume (MPV)、white blood cell count (WBC) and red blood cell count (RBC) *ex vivo*.

* p < 0.05 compared with the CMC-Na group.

To evaluate the ability of pimpinellin as an antiplatelet agent, platelet aggregation *ex vivo* was assessed. Pimpinellin produced a concentration-related inhibition of collagen-induced *ex vivo* aggregation in platelets, which was consistent with the inhibitory effect of pimpinellin on platelet aggregation *in vitro* (Figure 7C). These results suggested that pimpinellin inhibited platelet function under normal conditions *in vivo*.

We further evaluated the role of pimpinellin in hemostasis by measuring the tail bleeding time. We found that tail bleeding time was not affected in mice gavaged with low doses of pimpinellin (40 mg/kg) relative to the controls, while high doses of pimpinellin (100 mg/kg) prolonged the tail bleeding time (Figure 7D).

## Discussion

In recent years, traditional medicine has become more popular, and long-term clinical practice and scientific studies have shown that some herbs and their extracts have good antithrombotic and antiplatelet functions (Jin et al., 2007; Mousa, 2010; Lee et al., 2013; Gao et al., 2019). The active ingredient or drug monomer of a natural product is an important component for the development of new compounds. *Toddalia asiatica Lam* has a long history of medicinal use in China due to its various biological effects such as dispelling wind and pain, relieving bleeding, hemostasis and anti-inflammatory activities (Zhang et al., 2017; Alagaraj and Muthukrishnan, 2020). Pimpinellin is a coumarin-like compound extracted from *Toddalia asiatica Lam*. An understanding of the effects of pimpinellin and platelet function may help to develop improved strategies for the treatment of cardiovascular diseases. In this study, the antiplatelet effects of pimpinellin were analyzed.

We for the first time investigated the effects of pimpinellin on platelet aggregation, release, and spreading. The results indicated that pimpinellin inhibited collagen-induced platelet aggregation and reduced both dense and α granule secretion, and inhibited spreading and adhesion of human platelets. Besides, we further investigated the potential antiplatelet mechanism of pimpinellin, by focusing on the collagen/GPVI-mediated signaling pathway. The results demonstrated that pimpinellin may exert antiplatelet effects by inhibiting the PKC pathway as well as the PI3K-Akt-GSK3β/p38 MAPK pathway.

Pimpinellin significantly inhibited collagen-induced platelet aggregation, but did not affect thrombin- and ADP-induced platelet aggregation, suggesting that pimpinellin may has a specific inhibitory effect on collagen. We also found that pimpinellin was not toxic to platelets, and pimpinellin had no effect on LDH at concentrations that inhibited platelet aggregation.

Upon activation, platelets release the contents of three main types of granules: dense granules, alpha granules, and lysosomal granules (Blair and Flaumenhaft, 2009), which contain a variety of active substances. Dense granules and alpha granules play an important role in platelet aggregation and activation (Binsker et al., 2018). Besides, platelet dense granules contain ADP and polyphosphate, which contribute to hemostasis and coagulation. Alpha granules contain various growth factors and adhesion molecules P-selectin and CD63 (Fuentes and Palomo, 2013). Pimpinellin was found to inhibit collagen-induced ATP release and P-selectin expression. Exogenous supplementation of ADP partially reversed the inhibitory effect of pimpinellin on platelet aggregation. Pimpinellin exerted its inhibitory effect on platelet aggregation and activation by inhibiting the platelet release function.

Thromboxane A2 is produced by activated platelets during hemostasis and has procoagulant properties such as platelet activation and increased platelet aggregation (Fontana et al., 2014). However, TxA_2_ has a short half-life and TxB_2_ is produced by the non-enzymatic hydration of TxA_2_. Therefore, we measured the concentration of TxB_2_ to determine the level of TxA2 formation. Pimpinellin inhibited the formation of TxB_2_, suggesting that pimpinellin exerts its antiplatelet effect by inhibiting the formation of TxB_2_. cAMP is an important regulator of platelet activity and inhibits platelet aggregation induced by a variety of stimulants (Raslan and Naseem, 2014). Besides, cAMP is also involved in regulatory functions, such as calcium mobilization, adhesion, and skeletal rearrangement in platelets (Margarucci et al., 2011; Raslan et al., 2015). Our data showed that although pimpinellin tended to elevate cAMP levels, there was no significant *(p* > 0.05) difference compared to the control group.

When blood vessels are damaged, the exposed collagen substrate drives platelet activation and the formation of a hemostatic thrombus. The formation of the hemostatic thrombus results from continuous platelet adhesion, activation, and aggregation (Jurk and Kehrel, 2005; Mackman et al., 2007; Jurk and Kehrel, 2010). Platelet spreading is a process by which adherent platelets increase platelet contact with the damaged site after skeletal rearrangement at the site of thrombus damage, and this process is considered to be a critical step in hemostasis and thrombosis (Koupenova et al., 2018). In our study, we found that pimpinellin significantly (*p <* 0.05) reduced the number of platelet adhesions and average platelet spreading area. These results suggest that pimpinellin inhibits the adhesion and spreading function of platelets.

The platelet activation signals induced by specific receptors on platelets eventually converge into a common signaling event that induces an “inside-out” signaling process that activates integrin αIIbβ3 (Ginsberg, 2014). This leads to platelet spreading, secretion of additional granules, stabilization of platelet adhesion and aggregation, and clot retraction. Platelet adhesion to immobilized fibrinogen and mediated clot retraction involves an “outside-in” signaling and cytoskeletal reorganization of integrin αIIbβ3 (Hynes, 2002; Durrant et al., 2017). Clot retraction is an important process of vascular injury healing, and it also reflects the “outside-in” signal transduction process of platelets. Abnormal clot retraction indicates that the “outside-in” signal of platelets is damaged, and eventually leads to prolonged bleeding time and abnormal thrombus formation. We found that pimpinellin inhibited the binding of PAC-1 to activated integrin αIIbβ3, suggesting that pimpinellin inhibited the “inside-out” signaling. Pimpinellin also inhibited both platelets spreading area and clot retraction, suggesting that pimpinellin also inhibited “outside-in” signaling.

Many compounds or extracts of plant origin inhibited platelet function and mediated the collagen-induced GPVI signalling pathway. Glaucocalyxin A incubation reduced phosphorylation of SYK, LAT and PLCγ2 (Li et al., 2013); Tussilagone decreased the phosphorylation of Syk/PLCγ2-PKC/MAPK signaling pathways (Zhou et al., 2020); Salidroside inhibits platelet function through AKT/GSK3β signaling pathway (Wei et al., 2020). In the present study, we also demonstrated that pimpinellin inhibited multiple key protein phosphorylation related to collagen-induced signaling pathways in platelets. Activation of collagen/GPVI receptors leads to tyrosine phosphorylation of Fyn and Lyn, followed by Syk activation and LAT tyrosine phosphorylation (Poole et al., 1997), which in turn recruits PI3K and PLCγ2. PLCγ2 then catalyzes the release of DAG and IP3 from phosphatidylinositol for subsequent PKC activation and Ca^2+^ movement (Harper and Poole, 2010), ultimately leading to platelet secretion, aggregation, and thrombus formation. We found that pimpinellin inhibited Src416 (Supplementary Figure S3), Syk, PI3K, PLCγ2, and PKC phosphorylation. Therefore, our results suggest that pimpinellin inhibits collagen-stimulated platelet activation and aggregation by negatively regulating GPVI signaling.

We are familiar with the GPVI signals often involved in SYK, PLCγ2, SLP76 and PI3K(Watson et al., 2005). And phosphoinositide 3-kinase (PI3K) plays an important role in GPVI-mediated platelet activation (Chen et al., 2004; Gibbins, 2004; Kim et al., 2009). MAPKs, including ERK, p38 MAPK, and JNK, are also involved in collagen-induced platelet activation (Fan et al., 2018; Cargnello and Roux, 2011). p38 MAPK is reported to be a key signal for collagen-induced aggregation (Kuliopulos et al., 2004; Hanai et al., 2009). The present study showed that pimpinellin-mediated inhibition of collagen-stimulated platelet activation involves p38 MAPK activation but not ERK and JNK activation. It was demonstrated that PKCα not only affects the release of platelet alpha particles and dense granules (Konopatskaya et al., 2009), but also mediates the GPVI signaling pathway (Pula et al., 2005). Our data suggest that pimpinellin can effectively inhibit the phosphorylation of PKC. The PI3K/Akt signaling pathway regulates a variety of cellular processes such as inflammation, mitosis, and cell migration in several cell types (Fresno et al., 2004). In platelets, PI3K/Akt is an important regulator of platelet function such as granule secretion and activation of integrin αIIbβ3 (Niu et al., 2012). In this study, we found that pimpinellin inhibited collagen-induced phosphorylation of P85, Akt, and GSK3β (a recognized downstream effector protein of Akt). LY294002 is an inhibitor of the PI3K/Akt pathway and effectively reduces collagen-induced platelet aggregation and phosphorylation of Akt. Besides, we also found that a combination of pimpinellin and PI3K-specific inhibitor LY294002 (Vlahos et al., 1994), enhanced the inhibition of platelet aggregation by pimpinellin and Akt phosphorylation, suggesting that pimpinellin inhibition of platelet activation and function by inhibiting downstream of collagen-induced GPVI signaling downstream. We found that pimpinellin can affect the phosphorylation of SRC and Syk, thus suggesting that pimpinellin can affect GPVI proximal signaling.

In addition, we studied the effect of pimpinellin on the coagulation system. PT usually reflects the extrinsic coagulation system, while APTT is mainly used as an endogenous coagulation system (Liu et al., 2018). Pimpinellin (40 mg/kg) showed no effect on APTT and PT, suggesting that low doses of pimpinellin did not affect coagulation function. However, high doses of pimpinellin (100 mg/kg) significantly (*p* < 0.01) affected APTT, suggesting that the anticoagulation mechanism at high dose was mainly mediated by the endogenous coagulation system. The mice platelet *ex-vivo* assay also confirmed that pimpinellin (40 and 100 mg/kg) inhibited collagen-induced platelet aggregation. We also found that pimpinellin (40 mg/kg) did not affect tail bleeding time in mice, pimpinellin (100 mg/kg) prolonged tail bleeding in mice, suggesting that low doses of pimpinellin does not cause serious bleeding side effects at concentrations that inhibit platelet aggregation.

In conclusion, pimpinellin has significant anti-platelet aggregation, release, adhesion, and spreading effects and inhibits clot retraction. Besides, its anti-platelet effects are mainly mediated by key molecules in the collagen/GPVI signaling pathway, which involves Syk-SLP76-PLCγ2-PKC-p38 MAPK and PI3K/Akt signaling pathways. Our study shows that pimpinellin has anti-platelet activity in addition to its other well-known properties. This indicates that pimpinellin may be a potential agent for effectively preventing platelet-related thromboembolic diseases, and it may also be an ideal choice for preventing atherosclerotic diseases.

## Data Availability

The original contributions presented in the study are included in the article/supplementary material, further inquiries can be directed to the corresponding author.

## References

[B1] Alagaraj P. Muthukrishnan S. (2020). Toddalia Asiatica L. - A Rich Source of Phytoconstituents with Potential Pharmacological Actions, an Appropriate Plant for Recent Global Arena. Chamc 18 (2), 104–110. 10.2174/1871525718666200212095756 32048981

[B2] Binsker U. Palankar R. Wesche J. Kohler T. Prucha J. Burchhardt G. (2018). Secreted Immunomodulatory Proteins of *Staphylococcus aureus* Activate Platelets and Induce Platelet Aggregation. Thromb. Haemost. 47 (4), 745–757. 10.1055/s-0038-1637735 29554697

[B3] Blair P. Flaumenhaft R. (2009). Platelet α-granules: Basic Biology and Clinical Correlates. Blood Rev. 23 (4), 177–189. 10.1016/j.blre.2009.04.001 19450911 PMC2720568

[B4] Cargnello M. Roux P. P. (2011). Activation and Function of the MAPKs and Their Substrates, the MAPK-Activated Protein Kinases. Microbiol. Mol. Biol. Rev. 75 (1), 50–83. 10.1128/MMBR.00031-10 21372320 PMC3063353

[B5] Chen J. De S. Damron D. S. Chen W. S. Hay N. Byzova T. V. (2004). Impaired Platelet Responses to Thrombin and Collagen in AKT-1-Deficient Mice. Blood 104 (6), 1703–1710. 10.1182/blood-2003-10-3428 15105289 PMC1569945

[B6] Davì G. Patrono C. (2007). Platelet Activation and Atherothrombosis. N. Engl. J. Med. 357 (24), 2482–2494. 10.1056/NEJMra071014 18077812

[B7] Durrant T. N. van den Bosch M. T. Hers I. (2017). Integrin αIIbβ3 Outside-In Signaling. Blood 130 (14), 1607–1619. 10.1182/blood-2017-03-773614 28794070 PMC5672329

[B8] Faber D. R. de Groot P. G. Visseren F. L. J. (2009). Role of Adipose Tissue in Haemostasis, Coagulation and Fibrinolysis. Obes. Rev. 10 (5), 554–563. 10.1111/j.1467-789X.2009.00593.x 19460118

[B9] Fan X. Wang C. Shi P. Gao W. Gu J. Geng Y. (2018). Platelet MEKK3 Regulates Arterial Thrombosis and Myocardial Infarct Expansion in Mice. Blood Adv. 2 (12), 1439–1448. 10.1182/bloodadvances.2017015149 29941457 PMC6020810

[B10] Fontana P. Zufferey A. Daali Y. Reny J.-L. (2014). Antiplatelet Therapy: Targeting the TxA2 Pathway. J. Cardiovasc. Trans. Res. 7 (1), 29–38. 10.1007/s12265-013-9529-1 24353037

[B11] Fresno Vara J. A. Casado E. de Castro J. Cejas P. Belda-Iniesta C. González-Barón M. (2004). PI3K/Akt Signalling Pathway and Cancer. Cancer Treat. Rev. 30 (2), 193–204. 10.1016/j.ctrv.2003.07.007 15023437

[B12] Fuentes E. Palomo I. (2013). Relationship Between Platelet PPARs, cAMP Levels, and P-Selectin Expression: Antiplatelet Activity of Natural Products. Evidence-Based Complement. Altern. Med. 2013, 1–10. 10.1155/2013/861786 PMC3845334 24324520

[B13] Gallus A. S. Hirsh J. (1976). Antithrombotic Drugs. Drugs 12 (1), 41–68. 10.2165/00003495-197612010-00002 789043

[B14] Gao P. Li S. Liu K. Sun C. Song S. Li L. (2019). Antiplatelet Aggregation and Antithrombotic Benefits of Terpenes and Flavones from Hawthorn Leaf Extract Isolated Using the Activity-Guided Method. Food Funct. 10 (2), 859–866. 10.1039/c8fo01862f 30681694

[B15] Gibbins J. M. (2004). Platelet Adhesion Signalling and the Regulation of Thrombus Formation. J. Cel Sci. 117 (Pt 16), 3415–3425. 10.1242/jcs.01325 15252124

[B16] Ginsberg M. H. (2014). Integrin Activation. BMB Rep. 47 (12), 655–659. 10.5483/bmbrep.2014.47.12.241 25388208 PMC4345508

[B17] Hanai Y. Adachi S. Yasuda I. Takai S. Matsushima-Nishiwaki R. Kato H. (2009). Collagen-induced P38 MAP Kinase Activation Is a Biomarker of Platelet Hyper-Aggregation in Patients with Diabetes Mellitus. Life Sci. 85 (9-10), 386–394. 10.1016/j.lfs.2009.07.003 19631227

[B18] Harper M. T. Poole A. W. (2010). Diverse Functions of Protein Kinase C Isoforms in Platelet Activation and Thrombus Formation. J. Thromb. Haemost. 8 (3), 454–462. 10.1111/j.1538-7836.2009.03722.x 20002545

[B19] Hou S.-M. Hsia C.-W. Tsai C.-L. Hsia C.-H. Jayakumar T. Velusamy M. (2020). Modulation of Human Platelet Activation and In Vivo Vascular Thrombosis by Columbianadin: Regulation by Integrin αIIbβ3 Inside-Out but Not Outside-In Signals. J. Biomed. Sci. 27 (1), 60. 10.1186/s12929-020-0619-5 32375785 PMC7201758

[B20] Hu X.-L. Xu Z. Liu M.-L. Feng L.-S. Zhang G.-D. (2018). Recent Developments of Coumarin Hybrids as Anti-fungal Agents. Curr. Top. Med. Chem. 17 (29), 3219–3231. 10.2174/1568026618666171215100326 29243577

[B21] Huang J. Li X. Shi X. Zhu M. Wang J. Huang S. (2019). Platelet Integrin αIIbβ3: Signal Transduction, Regulation, and its Therapeutic Targeting. J. Hematol. Oncol. 12 (1), 26. 10.1186/s13045-019-0709-6 30845955 PMC6407232

[B22] Hynes R. O. (2002). Integrins. Cell 110 (6), 673–687. 10.1016/s0092-8674(02)00971-6 12297042

[B23] Jin L. Ying Z.-H. Yu C.-H. Zhang H.-H. Yu W.-Y. Wu X.-N. (2020). Isofraxidin Ameliorated Influenza Viral Inflammation in Rodents via Inhibiting Platelet Aggregation. Int. Immunopharmacology 84, 106521. 10.1016/j.intimp.2020.106521 32315950

[B24] Jin Y.-R. Yu J. Y. Lee J.-J. You S.-H. Chung J.-H. Noh J.-Y. (2007). Antithrombotic and Antiplatelet Activities of Korean Red Ginseng Extract. Basic Clin. Pharmacol. Toxicol. 100 (3), 170–175. 10.1111/j.1742-7843.2006.00033.x 17309520

[B25] Jorgensen L. (2006). The Role of Platelets in the Initial Stages of Atherosclerosis. J. Thromb. Haemost. 4 (7), 1443–1449. 10.1111/j.1538-7836.2006.02006.x 16839335

[B26] Jurk K. Kehrel B. E. (2010). Pathophysiologie und Biochemie der Thrombozyten. Internist 51 (9), 1086–1094. 10.1007/s00108-010-2595-4 20700569

[B27] Jurk K. Kehrel B. E. (2005). Platelets: Physiology and Biochemistry. Semin. Thromb. Hemost. 31 (4), 381–392. 10.1055/s-2005-916671 16149014

[B28] Kariuki H. Kanui T. Yenesew A. Patel N. Mbugua P. (2013). Antinocieptive and Anti-inflammatory Effects of Toddalia Asiatica (L) Lam. (Rutaceae) Root Extract in Swiss Albino Mice. Pan Afr. Med. J. 14, 133. 10.11604/pamj.2013.14.133.2130 23734278 PMC3670198

[B29] Kim S. Mangin P. Dangelmaier C. Lillian R. Jackson S. P. Daniel J. L. (2009). Role of Phosphoinositide 3-Kinase β in Glycoprotein VI-mediated Akt Activation in Platelets. J. Biol. Chem. 284 (49), 33763–33772. 10.1074/jbc.M109.048553 19700402 PMC2797145

[B30] Konopatskaya O. Gilio K. Harper M. T. Zhao Y. Cosemans J. M. E. M. Karim Z. A. (2009). PKCα Regulates Platelet Granule Secretion and Thrombus Formation in Mice. J. Clin. Invest. 119 (2), 399–407. 10.1172/JCI34665 19147982 PMC2631290

[B31] Koupenova M. Clancy L. Corkrey H. A. Freedman J. E. (2018). Circulating Platelets as Mediators of Immunity, Inflammation, and Thrombosis. Circ. Res. 122 (2), 337–351. 10.1161/CIRCRESAHA.117.310795 29348254 PMC5777300

[B32] Koupenova M. Kehrel B. E. Corkrey H. A. Freedman J. E. (2017a). Thrombosis and Platelets: An Update. Eur. Heart J. 38 (11), 785–791. 10.1093/eurheartj/ehw550 28039338 PMC11110018

[B33] Koupenova M. Kehrel B. E. Corkrey H. A. Freedman J. E. (2017b). Thrombosis and Platelets: An Update. Eur. Heart J. 38 (11), 785–791. 10.1093/eurheartj/ehw550 28039338 PMC11110018

[B34] Kuliopulos A. Mohanlal R. Covic L. (2004). Effect of Selective Inhibition of the P38 MAP Kinase Pathway on Platelet Aggregation. Thromb. Haemost. 92 (6), 1387–1393. 10.1160/TH04-03-0187 15583748

[B35] Lam M. Roszik J. Kanikarla-Marie P. Davis J. S. Morris J. Kopetz S. (2017). The Potential Role of Platelets in the Consensus Molecular Subtypes of Colorectal Cancer. Cancer Metastasis Rev. 36 (2), 273–288. 10.1007/s10555-017-9678-9 28681242

[B36] Lee J.-J. Kim T. Cho W.-K. Ma J. Y. (2013). Antithrombotic and Antiplatelet Activities of Soshiho-Tang Extract. BMC Complement. Altern. Med. 13, 137. 10.1186/1472-6882-13-137 23773779 PMC3686589

[B37] Li W. Tang X. Yi W. Li Q. Ren L. Liu X. (2013). Glaucocalyxin A Inhibits Platelet Activation and Thrombus Formation Preferentially via GPVI Signaling Pathway. PLoS One 8 (12), e85120. 10.1371/journal.pone.0085120 24386454 PMC3875551

[B38] Li X. Qiu Z. Jin Q. Chen G. Guo M. (2018). Cell Cycle Arrest and Apoptosis in HT-29 Cells Induced by Dichloromethane Fraction from Toddalia Asiatica (L.) Lam. Front. Pharmacol. 9, 629. 10.3389/fphar.2018.00629 29950999 PMC6008524

[B39] Liu G. Xie W. He A.-D. Da X.-W. Liang M.-L. Yao G.-q. (2016). Antiplatelet Activity of Chrysin via Inhibiting Platelet αIIbβ3-mediated Signaling Pathway. Mol. Nutr. Food Res. 60 (9), 1984–1993. 10.1002/mnfr.201500801 27006308

[B40] Liu J. Xu D. Xia N. Hou K. Chen S. Wang Y. (2018). Anticoagulant Activities of Indobufen, An Antiplatelet Drug. Molecules 23 (6), 1452. 10.3390/molecules23061452 PMC6099839 29914049

[B41] Liu Z. G. Wang X. Y. Mao B. P. Xie X. L. (2014). [Study on Chemical Constituents of Toddalia Asiatica]. Zhong Yao Cai 37 (9), 1600–1603. 10.13863/j.issn1001-4454.2014.09.024 25857161

[B42] Mackman N. Tilley R. E. Key N. S. (2007). Role of the Extrinsic Pathway of Blood Coagulation in Hemostasis and Thrombosis. Arterioscler Thromb. Vasc. Biol. 27 (8), 1687–1693. 10.1161/ATVBAHA.107.141911 17556654

[B43] Margarucci L. Roest M. Preisinger C. Bleijerveld O. B. van Holten T. C. Heck A. J. R. (2011). Collagen Stimulation of Platelets Induces a Rapid Spatial Response of cAMP and cGMP Signaling Scaffolds. Mol. Biosyst. 7 (7), 2311–2319. 10.1039/c1mb05145h 21597619

[B44] Michelson A. D. (2008). P2Y12Antagonism. Arterioscler Thromb. Vasc. Biol. 28 (3), s33–8. 10.1161/ATVBAHA.107.160689 18174449

[B45] Mousa S. A. (2010). Antithrombotic Effects of Naturally Derived Products on Coagulation and Platelet Function. Methods Mol. Biol. 663, 229–240. 10.1007/978-1-60761-803-4_9 20617421

[B46] Nilsson B. Back V. Wei R. Plane F. Jurasz P. Bungard T. J. (2019). Potential Antimigraine Effects of Warfarin: An Exploration of Biological Mechanism with Survey of Patients. TH Open 03 (2), e180–e189. 10.1055/s-0039-1692989 PMC6598089 31259301

[B47] Niu H. Chen X. Gruppo R. A. Li D. Wang Y. Zhang L. (2012). Integrin αIIb-Mediated PI3K/Akt Activation in Platelets. PLoS One 7 (10), e47356. 10.1371/journal.pone.0047356 23082158 PMC3474815

[B48] Poole A. Gibbins J. M. Turner M. van Vugt M. J. van de Winkel J. G. J. Saito T. (1997). The Fc Receptor γ-chain and the Tyrosine Kinase Syk Are Essential for Activation of Mouse Platelets by Collagen. EMBO J. 16 (9), 2333–2341. 10.1093/emboj/16.9.2333 9171347 PMC1169834

[B49] Pula G. Crosby D. Baker J. Poole A. W. (2005). Functional Interaction of Protein Kinase Cα with the Tyrosine Kinases Syk and Src in Human Platelets. J. Biol. Chem. 280 (8), 7194–7205. 10.1074/jbc.M409212200 15583006

[B50] Raslan Z. Aburima A. Naseem K. M. (2015). The Spatiotemporal Regulation of cAMP Signaling in Blood Platelets-Old Friends and New Players. Front. Pharmacol. 6, 266. 10.3389/fphar.2015.00266 26617518 PMC4639615

[B51] Raslan Z. Naseem K. M. (2014). The Control of Blood Platelets by cAMP Signalling. Biochem. Soc. Trans. 42 (2), 289–294. 10.1042/BST20130278 24646233

[B52] Rondina M. T. Weyrich A. S. Zimmerman G. A. (2013). Platelets as Cellular Effectors of Inflammation in Vascular Diseases. Circ. Res. 112 (11), 1506–1519. 10.1161/CIRCRESAHA.113.300512 23704217 PMC3738064

[B53] Stephen Irudayaraj S. Sunil C. Duraipandiyan V. Ignacimuthu S. (2012). Antidiabetic and Antioxidant Activities of Toddalia Asiatica (L.) Lam. Leaves in Streptozotocin Induced Diabetic Rats. J. Ethnopharmacol. 143 (2), 515–523. 10.1016/j.jep.2012.07.006 22842651

[B54] Vaiyapuri S. Roweth H. Ali M. S. Unsworth A. J. Stainer A. R. Flora G. D. (2015). Pharmacological Actions of Nobiletin in the Modulation of Platelet Function. Br. J. Pharmacol. 172 (16), 4133–4145. 10.1111/bph.13191 25988959 PMC4543618

[B55] Vlahos C. J. Matter W. F. Hui K. Y. Brown R. F. (1994). A Specific Inhibitor of Phosphatidylinositol 3-kinase, 2-(4-Morpholinyl)-8-Phenyl-4h-1-Benzopyran-4-One (LY294002). J. Biol. Chem. 269 (7), 5241–5248. 10.1016/s0021-9258(17)37680-9 8106507

[B56] Watson S. P. Auger J. M. Mccarty O. J. T. Pearce A. C. (2005). GPVI and Integrin alphaIIbbeta3 Signaling in Platelets. J. Thromb. Haemost. 3 (8), 1752–1762. 10.1111/j.1538-7836.2005.01429.x 16102042

[B57] Wei G. Xu X. Tong H. Wang X. Chen Y. Ding Y. (2020). Salidroside Inhibits Platelet Function and Thrombus Formation Through AKT/GSK3β Signaling Pathway. Aging 12 (9), 8151–8166. 10.18632/aging.103131 32352928 PMC7244060

[B58] Yue M. Luo D. Yu S. Liu P. Zhou Q. Hu M. (2016). Misshapen/NIK-related Kinase (MINK1) Is Involved in Platelet Function, Hemostasis, and Thrombus Formation. Blood 127 (7), 927–937. 10.1182/blood-2015-07-659185 26598717

[B59] Zaragozá C. Monserrat J. Mantecón C. Villaescusa L. Zaragozá F. Álvarez‐Mon M. (2016). Antiplatelet Activity of Flavonoid and Coumarin Drugs. Vasc. Pharmacol. 87, 139–149. 10.1016/j.vph.2016.09.002 27616636

[B60] Zhang X. Sun W. Yang Z. Liang Y. Zhou W. Tang L. (2017). Hemostatic Chemical Constituents from Natural Medicine Toddalia Asiatica Root Bark by LC-ESI Q-TOF MSE. Chem. Cent. J. 11 (1), 55. 10.1186/s13065-017-0283-3 29086834 PMC5472640

[B61] Zhou J. Yang R.-P. Song W. Xu H.-M. Wang Y.-H. (2020). Antiplatelet Activity of Tussilagone via Inhibition of the GPVI Downstream Signaling Pathway in Platelets. Front. Med. 7, 380. 10.3389/fmed.2020.00380 PMC7403204 32850895

