# Supplementary material for: Pimpinellin Inhibits Collagen-induced Platelet Aggregation and Activation Through Inhibiting Granule Secretion and PI3K/Akt Pathway
Source: Front Pharmacol. 2021 Jul 22;12:706363. doi: 10.3389/fphar.2021.706363 (PMC8339208; doi:10.3389/fphar.2021.706363)
Supplement: Supplementary file 1 [file DataSheet1.docx]

Supplementary Material

# ****

# **Supplementary** **Figure 1. Effects of pimpinellin on platelet aggregation .**

The human platelet-rich plasma (3 × 10^8^/mL) were incubated with different concentrations of pimpinellin (10μM and 20 μM) or the vehicle group for 5 minutes. Stimulated platelet aggregation with  ADP (3 μM and 5 μM).

**Supplementary Figure 2. HPLC chromatogram**

1. Chromatogram of standard pimpinellin 100μg/mL. (B) Chromatogram of 40mg/kg pimpinellin plasma sample by gavaged. (C) Chromatogram of 100mg/kg pimpinellin plasma sample by gavaged.

Experimental method:

1. The mice were divided into 2 groups; pimpinellin group (40 mg/kg and 100 mg/kg). To calculate the amount of gavage, mice were weighed and dose administration was performed by gavage daily for 1 week. Two hours after administration of the last gavage, mice blood was taken after anesthesia, and centrifuged at 1200 rpm for 10 minutes. The blood supernatant sample was diluted 1:5 (supernatant: acetonitrile). The supernatant was then centrifuged at 12000 rpm for 10 minutes, and the supernatant was passed through a microporous membrane and detected by high performance liquid chromatography.
2. The pimpinellin standard was prepared with acetonitrile at a concentration of 100μg/ml, and then checked by high performance liquid chromatography.
3. LC-100 high performance liquid chromatograph (Shanghai Wufeng), measured at 270nm. Detekteerimine 35 °C juures, liikuv faas metanool:vesi (70:30). Equipped with LC-P100plus binary high-pressure constant-flow pump, LC-UV100 plus UV detector, LC-CO100 column thermostat, LC-WS100 chromatography workstation , Diamonsil C18 (250 mm×4.6 mm, 5μm) analytical Chromatography Columns.

**Supplementary Figure 3. Inhibition of collagen-induced SRC phosphorylation by pimpinellin.**

Lysates in each group were analyzed by immunoblotting to determine the SRC416 phosphorylation levels. Band density was calculated by the Image J software. Bar graphs represent the mean ± SEM (n=3). **p* < 0.05 and ***p* < 0.01 compared to the vehicle group .

**Supplementary Figure 4. Thrombopoietin (TPO) levels of administered mice.**

Compared with the vehicle group, TPO plasma levels were significantly higher in mice gavaged with 100 mg/kg.Bar graphs represent the mean ± SEM (n=3). **p* < 0.05 compared to the vehicle group .

Experimental method:

The mice were randomly divided into 3 groups; pimpinellin group (40 mg/kg and 100 mg/kg) and vehicle group (0.5% CMC-Na). To calculate the amount of gavage, mice were weighed and dose administration was performed by gavage daily for 1 week. Two hours after administration of the last gavage, mice blood was taken after anesthesia, and centrifuged at 1000 rcf for 15 minutes. Supernatant was taken for testing.


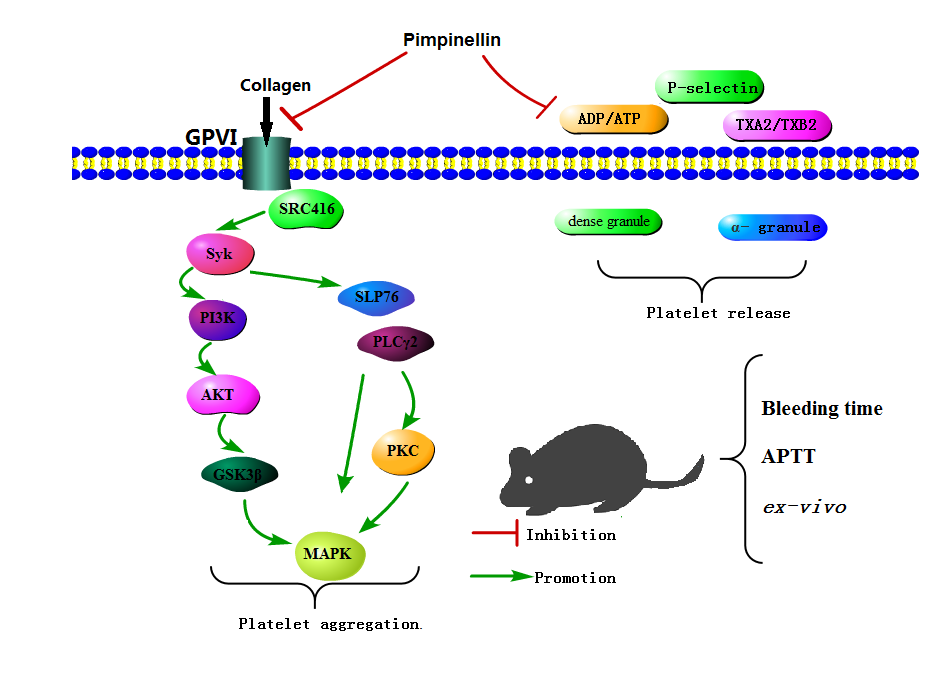


**Supplementary Material 5. Antiplatelet mechanism of pimpinellin。**
